# From Lab to Clinic: How Artificial Intelligence (AI) Is Reshaping Drug Discovery Timelines and Industry Outcomes

**DOI:** 10.3390/ph18070981

**Published:** 2025-06-30

**Authors:** Doni Dermawan, Nasser Alotaiq

**Affiliations:** 1 Department of Applied Biotechnology, Faculty of Chemistry, Warsaw University of Technology, 00-661 Warsaw, Poland; doni.dermawan.stud@pw.edu.pl; 2 Health Sciences Research Center (HSRC), Imam Mohammad Ibn Saud Islamic University (IMSIU), Riyadh 13317, Saudi Arabia

**Keywords:** artificial intelligence, clinical outcomes, clinical trials, drug discovery, hit identification, lead optimization, pharmaceutical industry

## Abstract

**Background/Objectives:** Artificial intelligence (AI) is transforming drug discovery and development by enhancing the speed and precision of identifying drug candidates and optimizing their efficacy. This review evaluates the application of AI in various stages of drug discovery, from hit identification to lead optimization, and its impact on clinical outcomes. The objective is to provide insights into the role of AI across therapeutic areas and assess its contributions to improving clinical trial efficiency and pharmaceutical outcomes. **Methods:** A systematic review followed PRISMA guidelines to analyze studies published between 2015 and 2025, focusing on AI in drug discovery and development. A comprehensive search was performed across multiple databases to identify studies employing AI techniques. The studies were categorized based on AI methods, clinical phase, and therapeutic area. The percentages of AI methods used, clinical phase stages, and the therapeutic regions were analyzed to identify trends. **Results:** AI methods included machine learning (ML) at 40.9%, molecular modeling and simulation (MMS) at 20.7%, and deep learning (DL) at 10.3%. Oncology accounted for the majority of studies (72.8%), followed by dermatology (5.8%) and neurology (5.2%). In clinical phases, 39.3% of studies were in the preclinical stage, 23.1% in Clinical Phase I, and 11.0% in the transitional phase. Clinical outcome reporting was observed in 45% of studies, with 97% reporting industry partnerships. **Conclusions:** AI significantly enhances drug discovery and development, improving drug efficacy and clinical trial outcomes. Future work should focus on expanding AI applications into underrepresented therapeutic areas and refining models to handle complex biological systems.

## 1. Introduction

The traditional drug discovery and development process is an arduous and resource-intensive endeavor. Historically, it takes approximately 10 to 15 years to develop a new therapeutic agent from initial discovery to regulatory approval, with costs often exceeding USD 1 to USD 2 billion [1,2,3]. Moreover, the likelihood of a new compound successfully navigating all phases of clinical trials and reaching the market remains dismally low, and estimates suggest that fewer than 1 in 10 drug candidates entering Phase I clinical trials are ultimately approved [4,5]. Key bottlenecks include the inefficient identification of druggable targets, the costly and time-consuming high-throughput screening (HTS) of large chemical libraries, suboptimal lead optimization, and poorly designed clinical trials that fail to stratify patient populations adequately [6]. Artificial Intelligence (AI) has emerged as a pivotal tool in the pharmaceutical and biomedical industries in recent years. By leveraging massive datasets, advanced algorithms, and high-performance computing, AI tools can uncover patterns and insights that would be nearly impossible for human researchers to detect unaided. These tools have been applied to almost any stage of the drug discovery pipeline, from target identification and validation, hit-to-lead optimization, absorption, distribution, metabolism, excretion, and toxicity (ADMET) profiling, to clinical trial simulation and recruitment strategies [7,8]. Unlike traditional, largely sequential workflows, AI models can parallel process and integrate multi-omics data streams (genomic, proteomic, phenotypic, chemical), potentially compressing the preclinical phase from several years to a few months [9,10].

Several AI-native and AI-integrated biotech firms have already demonstrated tangible progress in reducing timelines and increasing efficiency. Insilico Medicine, a leading AI drug discovery company, announced in 2021 that it successfully identified a novel target for idiopathic pulmonary fibrosis and advanced a drug candidate into preclinical trials in just 18 months (a process that typically takes 4–6 years) at a cost of only USD 150,000, excluding wet lab validation [11]. Similarly, Exscientia, in partnership with Sumitomo Dainippon Pharma, developed a novel small-molecule drug candidate (DSP-1181) for obsessive–compulsive disorder (OCD) in less than 12 months, making it the first AI-designed molecule to enter human clinical trials [12]. Another prominent example, Recursion Pharmaceuticals, uses automated high-throughput imaging combined with deep learning (DL) models to identify phenotypic changes in cells, allowing for rapid repurposing of existing molecules and discovering novel therapeutics [13]. Meanwhile, Schrödinger, renowned for its physics-based molecular simulations, integrates AI to predict molecular interactions with high accuracy. This hybrid approach of physics-informed AI is revolutionizing virtual screening by significantly improving hit rates and reducing reliance on exhaustive laboratory testing [14]. These pioneering firms are not just proof-of-concept models; they represent a paradigm shift in how drugs are discovered, optimized, and brought to market. Increasing investment from big pharmaceutical companies (e.g., Roche, Bayer, Novartis) into AI collaborations further underscores the seriousness with which the industry views these innovations. Despite these remarkable advances, the extent of AI’s measurable impact on key drug development metrics, such as preclinical cycle times, clinical success rates, and regulatory approval efficiencies, remains poorly characterized in the literature. While individual case studies and company press releases highlight success stories, there is a pressing need for a systematic, evidence-based review that consolidates current findings and evaluates the robustness of AI’s contributions across different stages of the drug development lifecycle. Furthermore, understanding these AI platforms’ scalability, reproducibility, and generalizability is critical for stakeholders, including regulators, investors, and clinical researchers, who are cautiously optimistic but demand empirical validation before widespread adoption.

This systematic review aimed to address the existing knowledge gap by comprehensively analyzing published literature on the application of AI in drug discovery and development. The primary objective was to evaluate how AI influenced the acceleration of drug development timelines, particularly from target identification through preclinical optimization and into clinical trials. The review quantified the impact of AI tools on critical performance metrics such as the reduction in development cycle times, improvements in hit identification and lead optimization, and the readiness for Investigational New Drug (IND) applications. Secondary objectives included assessing the enhancement of pipeline productivity, time-to-IND milestones, and real-world clinical outcomes of drug candidates developed or optimized using AI. Furthermore, the review examined broader industry trends, including licensing agreements, venture capital investments, strategic partnerships, and major pharmaceutical companies’ incorporation of AI frameworks. By synthesizing these findings, the review provided meaningful insights into AI’s practical impact on the pharmaceutical industry and offered evidence to guide future research directions, regulatory considerations, and commercial strategies.

## 2. Results

### 2.1. Systematic Literature Search and Study Selection Workflow

**Figure 1:**
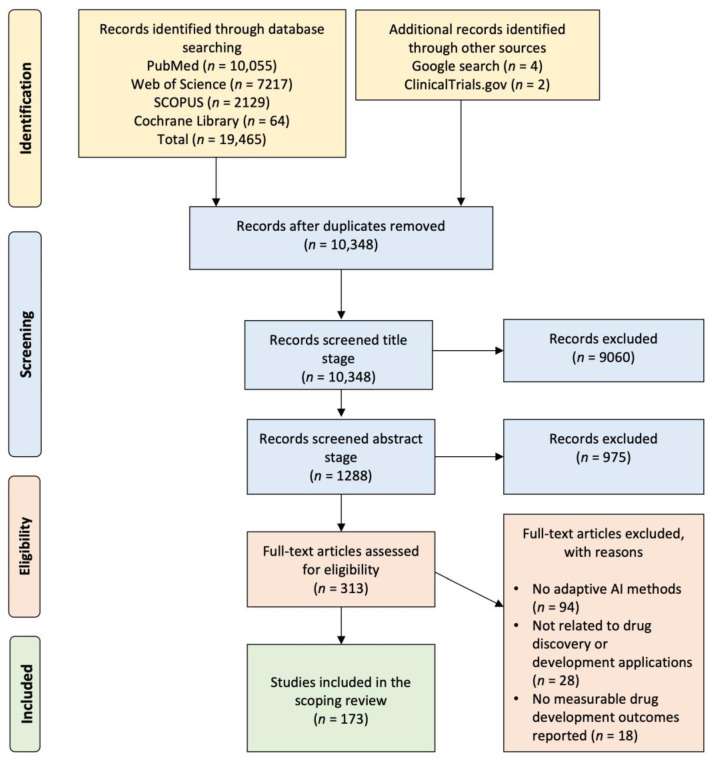
PRISMA (preferred reporting items for systematic reviews and meta-analyses) flow diagram of study selection process. The diagram illustrates how records were identified, screened, excluded, and ultimately included in the systematic review, following PRISMA guidelines.

A systematic literature search was conducted across four major scientific databases to capture a comprehensive set of studies involving AI in drug discovery and development. The initial search phase retrieved a total of 19,465 records. Among these, the highest number of records originated from PubMed (*n* = 10,055, representing 51.6% of all records), followed by Web of Science (*n* = 7217, or 37.1%), SCOPUS (*n* = 2129, or 10.9%), and a smaller number from the Cochrane Library (*n* = 64, or 0.3%) (Figure 1). In addition, six additional records were identified through manual searches and gray literature sources, including four from Google search and two from ClinicalTrials.gov, which together contributed only 0.03% to the total count. After removing duplicates across these sources, 10,348 unique records were retained for further screening, indicating that 46.9% of the initially identified articles were duplicates. These 10,348 records then underwent a title screening phase to evaluate fundamental relevance to the topic. At this stage, 9060 records (equivalent to 87.6% of the screened titles) were excluded due to being unrelated to AI applications in drug development or lacking relevance to pharmaceutical sciences. The remaining 1288 records (12.4% of those screened by title) proceeded to the next stage: abstract screening.

A more refined selection process was carried out during the abstract screening phase. Each abstract was reviewed for the use of AI techniques in the context of drug discovery or development and the presence of meaningful scientific or therapeutic contributions. As a result, 975 records (75.7% of abstracts reviewed) were excluded because they did not meet these criteria. The remaining 313 articles (24.3% of abstracts screened; 1.6% of the original dataset) were retained for full-text review. The full-text review phase involved a detailed assessment of the study’s methodology, focus, and outcomes. From these 313 full-text articles, 140 articles (44.7%) were excluded. The reasons for exclusion were systematically categorized as follows: 94 articles (30.0% of full texts assessed; 67.1% of exclusions) did not utilize adaptive AI methods, such as reinforcement learning (RL), generative adversarial networks, or other self-improving algorithms, which were key inclusion criteria. Twenty-eight articles (8.9% of full texts; 20.0% of exclusions) were unrelated to drug discovery or development applications. These included AI use in other domains such as medical diagnostics or image analysis, without clear links to pharmaceutical pipelines. Eighteen articles (5.8% of full texts; 12.9% of exclusions) lacked measurable drug development outcomes, such as predictive performance, lead optimization, or translational results, rendering them insufficiently aligned with the review objectives. Following this rigorous filtering process, 173 studies were deemed eligible and subsequently included in the final scoping review. This constitutes 55.3% of the full-text articles assessed, and only 1.8% of the total records identified at the outset. Despite the relatively small final inclusion rate, this subset of studies provides a focused and high-quality evidence base for analyzing how adaptive AI methods are currently integrated within the pharmaceutical research and development landscape. These selected studies form the basis of the subsequent analysis on methodological trends, therapeutic targets, and measurable impacts of AI in modern drug discovery.

### 2.2. Distribution of AI Applications Across Drug Development Stages, Geographic Trends, Industry Collaboration, and AI Technology Adoption

**Figure 2:**
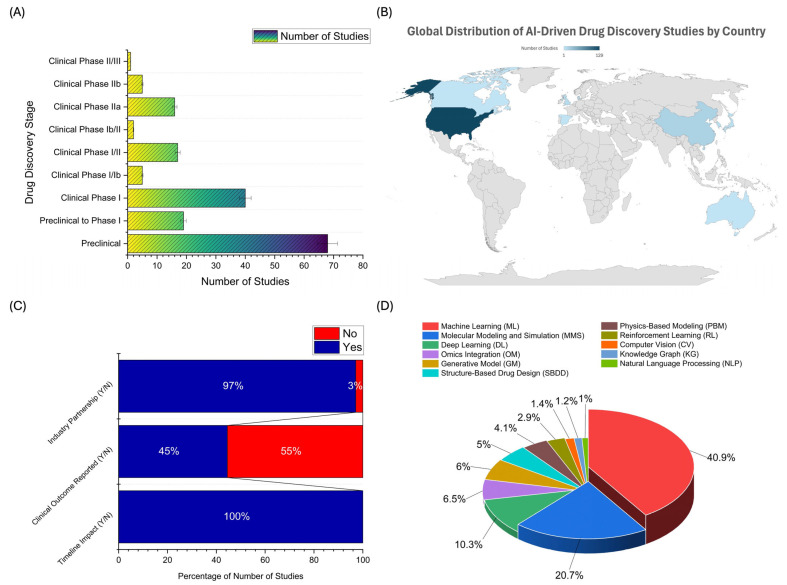
Comprehensive analysis of AI-driven drug discovery studies: distribution across drug development stages, global research activity by country, industry collaboration, clinical reporting, and utilization of AI methodologies. (**A**) A horizontal bar graph showing the number of AI-driven drug discovery studies categorized by their developmental stage, from preclinical research through various phases of clinical trials. (**B**) A world map indicating the number of AI-driven drug discovery studies conducted per country. (**C**) A series of bar graphs illustrating the percentage of studies reporting (1) industry partnerships, (2) clinical outcomes, and (3) translational impact. (**D**) A pie chart depicting the proportion of various AI technologies used in the studies. ML and DL dominate the field, accounting for 40.9% and 20.7% of studies, respectively.

A comprehensive analysis of the 173 included studies reveals valuable insights into how AI is applied throughout various stages of drug development, its geographic distribution, the degree of industry partnership, translational impact, and the spectrum of AI methodologies employed. Figure 2A provides a comprehensive overview of the distribution of AI applications across various stages of drug development, with an intense concentration in the early phases of the drug discovery pipeline. The preclinical stage remains the most active, with 68 out of 173 studies (39.3%) focused at this level. This highlights AI’s integral role in the foundational aspects of drug research, including target identification, virtual screening, de novo molecule generation, molecular docking, quantitative structure–activity relationship (QSAR) modeling, and ADMET prediction. The dominance of preclinical AI applications underscores its transformative potential in accelerating early-stage discovery and optimizing lead compounds before entering human trials. Next, 19 studies (11.0%) were positioned in the transitional phase between preclinical and Clinical Phase I, often associated with IND (Investigational New Drug) enabling activities. At this stage, AI is applied in predictive toxicology, in silico dose selection, early biomarker discovery, and simulation of pharmacokinetic parameters, contributing to safer and more informed transitions into human testing.

The Clinical Phase I stage showed a significant increase in AI integration, with 40 studies (23.1%), indicating growing confidence in AI tools for safety profiling, pharmacokinetics modeling, and real-time monitoring during early human exposure. These tools assist in optimizing dosage regimens and reducing early-phase attrition by predicting adverse events and guiding early clinical decisions. Five studies (2.9%) were conducted at the Clinical Phase I/Ib level and 17 studies (9.8%) at the Phase I/II level in the hybrid early clinical stages. These phases typically involve dose expansion and initial efficacy assessments, where AI supports patient stratification, digital biomarker identification, and adaptive trial designs. Moving into Clinical Phase Ib/II and Phase IIa, the number of AI-based studies declines, with 2 (1.2%) and 16 (9.2%) studies, respectively. These phases often focus on proof-of-concept evaluation, and AI is leveraged here to analyze multidimensional patient data, integrate omics datasets, and assist in go/no-go decisions. Despite the complexity of these phases, the use of AI remains underutilized. The later stages of development show the lowest AI engagement: Clinical Phase IIb with five studies (2.9%) and Clinical Phase II/III with just two studies (0.6%). These phases are centered on dose optimization, large-scale efficacy, and comparative effectiveness trials, where high regulatory standards, data integrity demands, and the need for explainability and validation of AI models limit the application of AI.

Figure 2B illustrates the global geographic distribution of AI-based drug discovery studies, revealing a highly skewed landscape dominated by a few technologically advanced nations. The United States stands at the forefront, contributing 129 out of 179 studies (72.1%), underscoring its leading role in the convergence of AI and pharmaceutical innovation. This dominance is attributed to multiple factors, including robust federal and private sector investment in biotechnology, an extensive network of academic and industry collaborations, high-performance computing infrastructure, and an entrepreneurial ecosystem that fosters the rapid development and deployment of AI platforms in drug discovery. However, the aim here is not to evaluate individual digital ecosystems or AI platforms but to characterize how national-level strategies influence broader trends in AI integration across the drug development continuum. In a distant second, China contributed 16 studies (8.9%), reflecting its rapidly expanding capabilities in AI and pharmaceutical research and development (R&D). China’s significant government support for AI innovation, coupled with its growing pharmaceutical market and increasing academic output in computational biology, signals strong momentum in this space. The United Kingdom accounted for nine studies (5.0%), indicative of its established strength in biomedical research, AI innovation hubs in Cambridge, London, and Oxford, and a regulatory environment that supports digital health technologies. Other contributors include South Korea (6 studies; 3.4%), Denmark (6 studies; 3.4%), and Japan (6 studies; 3.4%). These countries possess strong healthcare systems, government-sponsored AI initiatives, and active collaborations between academic and pharmaceutical sectors. Their participation reflects growing regional investment in AI-assisted drug development, albeit at a more modest scale compared to the US. More minor but notable contributions were seen from Singapore (3 studies; 1.7%), Spain (2 studies; 1.1%), Canada (1 study; 0.6%), and Australia (1 study; 0.6%). These nations are increasingly integrating AI into biomedical research but may face challenges related to scale, funding, or data accessibility that limit broader adoption. Notably, regions such as Africa, South America, and large parts of Southeast Asia were absent from the dataset, highlighting a significant global disparity in adopting and implementing AI technologies in drug discovery. This absence underscores the need for capacity-building initiatives, enhanced international collaborations, and knowledge-sharing frameworks that can democratize access to AI resources and training.

Figure 2C provides a comprehensive assessment of the translational maturity of AI-driven drug discovery studies, focusing on three critical indicators: timeline impact, clinical outcome reporting, and industry partnership. Remarkably, all 173 studies (100%) demonstrated some form of timeline impact, signifying that AI integration consistently contributes to accelerating various stages of the drug development pipeline. This universal influence highlights AI’s role in enhancing research efficiency, expediting compound selection, and shortening time-to-decision in early-stage drug discovery. A prominent example is Insilico Medicine’s AI-designed drug candidate INS018_055, a small molecule targeting idiopathic pulmonary fibrosis (IPF) [15]. Developed using their proprietary AI platform, this compound progressed from target identification to a preclinical candidate in under 18 months—a process that traditionally takes several years. As of recent updates, INS018_055 has entered Phase II clinical trials, underscoring how AI can dramatically compress timelines and bridge computational discovery with clinical translation. However, when examining clinical outcome reporting, the data reveals a significant drop-off in translational depth. Only 77 studies (45%) reported measurable clinical outcomes, such as pharmacokinetic parameters, safety profiles, or preliminary efficacy indicators. The majority of 96 studies (55%) did not report any clinical outcomes, suggesting that many AI applications remain confined to preclinical or in silico stages, where direct clinical relevance is either unexplored or not yet achieved. This imbalance highlights a key bottleneck in validating AI-derived insights within real-world clinical contexts. Furthermore, formal industry partnerships were surprisingly high, with 168 studies (97%) reporting collaboration with industry stakeholders. This finding contrasts with prior expectations and suggests that AI in drug discovery is increasingly seen as a strategic asset by pharmaceutical and biotech companies. These partnerships likely facilitate access to proprietary datasets, advanced computational tools, and regulatory expertise, which can enhance AI solutions’ real-world applicability and scalability.

Industry-wide licensing agreements and strategic collaborations have become primary conduits for integrating AI into mainstream pipelines. For instance, Sanofi entered into a USD 1.2 billion partnership with Exscientia to use AI to discover novel oncology and immunology therapies [16]. Similarly, AstraZeneca has formed long-term collaborations with BenevolentAI, embedding AI platforms directly into its discovery engine [17]. These high-value deals validate the commercial potential of AI-driven discovery and position pharmaceutical companies to leverage AI across multiple therapeutic areas. From a funding perspective, the sector is also witnessing substantial venture capital investment. In 2023 alone, AI-driven biotech startups raised over USD 4.5 billion globally, with major recipients including Recursion Pharmaceuticals, XtalPi, and Insilico Medicine. Recursion, in particular, has established an expansive AI-first infrastructure integrating single-cell analysis, phenotypic imaging, and multi-modal data fusion, attracting public market interest (IPO in 2021) and private capital from major players such as SoftBank [18]. Big Pharma continues to gain momentum in the incorporation of AI frameworks. Pfizer, Merck, Roche, and Novartis have each developed or partnered on bespoke AI initiatives. Pfizer, for example, uses IBM Watson and other internal models for clinical trial optimization and target prediction [19]. Roche acquired Flatiron Health and Foundation Medicine to strengthen its data analytics and real-world evidence platforms, reinforcing AI’s role in personalized oncology [20]. Meanwhile, Novartis has embedded AI into its Novartis AI Innovation Lab, leveraging partnerships with Microsoft to create scalable machine learning (ML) frameworks for drug discovery and development [21].

Figure 2D provides an in-depth overview of the diverse AI techniques employed across drug discovery studies, revealing a dynamic and evolving technological landscape. ML remains the most dominant technique, featured in 170 studies (40.9%), representing a significant proportion of the total. Its prevalence reflects ML’s broad utility in tasks such as predictive modeling, compound prioritization, toxicity forecasting, and high-dimensional data interpretation. ML’s adaptability and scalability make it a cornerstone of AI-enabled drug discovery platforms [22]. Molecular modeling and simulation (MMS) was the second most frequently applied method in 86 studies (20.7%). MMS techniques, such as molecular docking, molecular dynamics simulations, and free energy perturbation, are fundamental to structure-based drug design and offer mechanistic insights that complement data-driven approaches [23,24,25]. DL, used in 43 studies (10.3%), underscores the growing reliance on complex neural networks for handling unstructured data such as molecular images, protein structures, and compound libraries. DL techniques are particularly advantageous in modeling nonlinear relationships and developing end-to-end learning frameworks, including convolutional and graph neural networks that power many modern cheminformatics and bioinformatics pipelines [26,27]. Omics integration (OM) was observed in 27 studies, reflecting the increasing emphasis on multi-omics data (genomics, proteomics, metabolomics) for systems-level understanding of disease mechanisms and drug response. Generative models (GM) appeared in 25 studies, illustrating the rising interest in de novo molecular generation. These models, which include variational autoencoders (VAEs), generative adversarial networks (GANs), and transformer-based frameworks, are instrumental in creating novel chemical entities with optimized pharmacokinetic and pharmacodynamic profiles [28,29]. Their use highlights the shift toward AI systems capable not only of prediction but also of creativity.

Structure-based drug design (SBDD) techniques were used in 21 studies, emphasizing their role in rational drug design informed by high-resolution structural data. SBDD often integrates docking, pharmacophore modeling, and homology modeling, which, combined with AI tools, accelerate the discovery of structure–activity relationships [30,31]. Physics-based modeling (PBM) was utilized in 17 studies, combining AI with traditional biophysical simulations to capture atomic-level interactions and predict binding affinities with higher precision. RL was employed in 12 studies, gradually adopting this powerful paradigm for optimizing compound synthesis paths, multi-objective design, and adaptive trial strategies. RL’s ability to learn optimal policies from sequential decisions makes it especially suitable for iterative drug design workflows [32,33]. Computer vision (CV) techniques, found in six studies, were mainly applied in analyzing histopathological images, high-throughput screening outputs, and chemical structure recognition [34]. Despite being a relatively underutilized method, CV has strong potential in visual data processing across drug discovery and diagnostics. Knowledge graphs (KG) were used in five studies, facilitating the integration of heterogeneous biomedical data into relational frameworks. KGs enable AI systems to infer novel relationships, such as drug-disease or gene-compound links, supporting hypothesis generation and drug repurposing [35,36]. Natural language processing (NLP) appeared in four studies, primarily focused on mining scientific literature, clinical trial records, and patents for actionable insights [37,38]. NLP tools are pivotal in automating knowledge extraction and transforming unstructured text into structured datasets for AI models.

**Figure 3:**
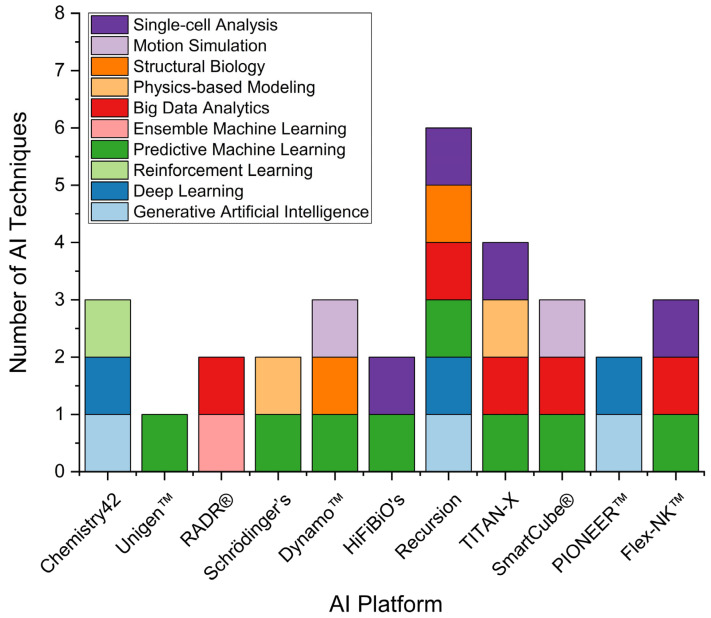
Diversity of AI techniques utilized across leading AI drug discovery platforms. This figure presents a comparative analysis of the number and variety of AI techniques implemented by major AI platforms in drug discovery. Each bar represents a distinct AI platform, and each colored segment within the bars corresponds to a specific AI technique.

Figure 3 highlights the diversity and technological sophistication of leading AI drug discovery platforms by illustrating the number and types of AI techniques integrated into each. Each bar represents a prominent platform developed by a pharmaceutical or AI-driven biotech company, and the colored segments within each bar denote the variety of AI approaches implemented. This comparative analysis offers a bird’s-eye view of innovation trends and technological focus areas within the AI-biopharma ecosystem. As a proxy for success, the platforms discussed are primarily evaluated based on their reported advancement of AI-supported drug candidates into preclinical or clinical development phases. Statements regarding AI functionality are derived from company disclosures, peer-reviewed publications, or white papers, and are acknowledged as such where independent validation is unavailable. Chemistry42, developed by Insilico Medicine, is reported to integrate generative AI, DL, and RL, three core methods that enable the platform to autonomously design novel chemical structures and optimize them for drug-like properties. This configuration supports an end-to-end generative chemistry pipeline, where molecules are created and refined iteratively through RL. Although it does not incorporate big data analytics or structural biology integration, Insilico positions Chemistry42 as a focused tool for early-stage molecular design [39].

Unigen™, from Compugen, stands out for its singular focus on predictive ML. Although it does not yet integrate other AI techniques, this focus enables it to excel in structure prediction, synthesis planning, and molecular property forecasting. This claim is based on company-reported use cases and reflects a streamlined strategy centered on quantum mechanics and in silico modeling. RADR^®^ AI, the platform developed by Lantern Pharma, represents a different approach. It uses ensemble ML and big data analytics, reflecting its strong orientation toward biomarker discovery, drug repurposing, and precision oncology [40]. Rather than generating new chemical entities, RADR^®^ reportedly analyzes existing compounds and omics datasets to identify therapeutic matches. This reflects a broader trend in AI-facilitated drug repositioning and personalized therapy development. Schrödinger’s platform integrates predictive ML with physics-based modeling, where AI algorithms are used to learn from quantum mechanical simulations and molecular dynamics data to accelerate binding affinity prediction and optimize ligand-protein interactions. Rather than replacing physics-based models, AI enhances these simulations by guiding conformational sampling, predicting simulation outcomes, and refining scoring functions for virtual screening. This hybrid approach reinforces Schrödinger’s role as a leader in structure-guided drug design, simulating molecular interactions at the atomic level while grounding predictions in physical and chemical reality. Dynamo™, by Relay Therapeutics, also exemplifies this integration by combining predictive ML, motion simulation, and structural biology. In this context, AI is employed to interpret the dynamic conformational landscapes generated by molecular simulations, enabling the identification of cryptic pockets and transient binding sites relevant for drug targeting. The AI layer thus transforms raw simulation data into actionable drug design insights.

Platforms like HiFiBiO’s DIS™ and Flex-NK™ showcase the specialized application of AI in immunotherapy and cell-based therapies. HiFiBiO focuses on single-cell analysis and predictive ML, enabling the discovery of immune targets at a granular resolution, which is ideal for antibody and T-cell engineering. Flex-NK™, on the other hand, leverages DL, predictive ML, and single-cell analysis to optimize NK-cell-based cancer therapies, highlighting the growing role of AI in cellular and gene therapy design. Recursion OS leads the field regarding AI technique integration, employing six out of ten tracked AI approaches: generative AI, DL, predictive ML, big data analytics, structural biology, and single-cell analysis. This diversity reflects Recursion’s commitment to large-scale phenotypic screening and multi-modal data fusion. The platform analyzes vast amounts of image-based cellular data and integrates it with molecular insights to drive target discovery and drug repurposing. This capability is inferred mainly from corporate communications and published output, as with other platforms. TITAN-X, developed by the NIMML Institute, emphasizes protein engineering by combining predictive ML algorithms with structural biology and physics-based simulations. Here, AI assists in predicting folding patterns, protein stability, and evolutionary trajectories by learning from structural datasets and simulation results. This AI-physics synergy is particularly powerful for designing synthetic proteins with tailored functionalities. SmartCube^®^, in contrast, incorporates motion simulation, predictive ML, and big data analytics to study behavioral phenotypes, particularly in neuropsychiatric disorders, a niche application of AI that blends behavioral science with drug discovery [41]. Lastly, PIONEER™ by Evaxion Biotech showcases a compact but impactful setup with generative AI and DL, aligning with Evaxion Biotech’s mission to build human-centric drug development platforms. This platform personalizes translational research, particularly where patient heterogeneity influences therapeutic outcomes [42]. It uses digital twin technology and AI to enhance translational research, especially in areas where patient variability significantly affects outcomes.

### 2.3. Landscape of AI Applications in Pharmaceutical R&D: Trends and Case Studies

Over the past decade (2015–2025), the pharmaceutical industry has witnessed a transformative integration of AI across drug discovery and development pipelines. Table 1 provides a comprehensive overview of AI-enabled innovations deployed across various therapeutic domains, ranging from oncology and gastroenterology to infectious diseases and immunology. Oncology emerges as the most prominent therapeutic area leveraging AI tools. Multiple case studies from biotech firms like A2A Pharmaceuticals, Accutar Biotechnology, Black Diamond Therapeutics, Compugen, and Evaxion Biotech highlight the versatile use of AI in structure-based drug design, epitope prediction, predictive modeling, and virtual screening. For instance, a study utilizes structure-based drug design combined with virtual screening and ADMET predictions to target triple-negative breast cancer (TNBC), high-grade serous ovarian cancer (HGSOC), and endometrial cancer with TP53 mutations [43]. Similarly, another study employed AI platforms such as ChemiRise and Chemi-Net to optimize the pharmacokinetics/pharmacodynamics (PK/PD) of potential drugs for ER+/HER2− breast cancer [44]. The consistent clinical advancement into Phase I and II trials underscores AI’s clinical promise in oncologic settings. Beyond target identification, AI is also used for combination therapy modeling and antibody design. Platforms like Unigen™, NovareAI, and Flex-NK™ integrate ML with omics data and spatial transcriptomics to identify novel therapeutic strategies. Exscientia and Evotec, for instance, utilize generative design models and simulation-guided clinical trial planning, marking a shift from empirical to computationally guided therapeutic development.

While oncology dominates, AI’s impact extends to a broad spectrum of other therapeutic areas. In gastroenterology, Celsius Therapeutics and Landos Biopharma use platforms like SCOPE and TITAN-X for single-cell RNA-seq analysis, predictive analytics, and disease stratification in conditions like ulcerative colitis and Crohn’s disease. In infectious diseases, Generate Biomedicines applied iterative ML-based chemoproteomic loops for COVID-19 prophylaxis, while Drug Farm’s IDInVivo platform enabled in vivo gene targeting for Hepatitis B. In neurology and immunology, platforms such as Magellan™ (Gain Therapeutics) and DeepCure’s AI-driven compound design system illustrate how AI is pushing the frontier of precision medicine. In rheumatology, Formation Bio used AI to automate MRI segmentation and analyze cartilage degradation in osteoarthritis. Such examples show how AI is revolutionizing both molecular-level innovations and system-level clinical decision-making processes. The diversity of AI techniques employed across these case studies reflects the maturity and versatility of the field. Methods range from knowledge graphs and Bayesian modeling to convolutional neural networks and RL. For example, a study leveraged knowledge graphs for target prioritization in ulcerative colitis [47], while another study used generative AI and RL for chemical space exploration in rheumatoid arthritis [56]. Integration with supercomputing resources, as seen in BERG’s use of the Oak Ridge supercomputer, and the construction of AI-driven lipid nanoparticle optimization (AiLNP) platforms by METiS Pharmaceuticals, exemplify cross-disciplinary innovation between AI, physics, and pharmaceutical sciences.

**Figure 4:**
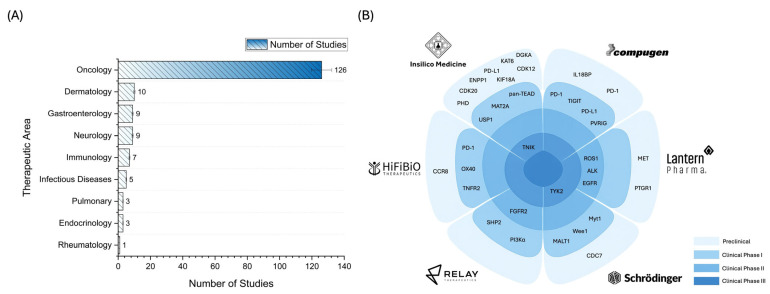
Therapeutic focus and molecular target distribution in AI-driven drug discovery. (**A**) Bar chart showing the number of studies across various therapeutic areas. Oncology dominates with 126 studies, followed by dermatology (10), gastroenterology (9), neurology (9), and immunology (7), indicating a primary focus on cancer treatment in AI-based drug development. (**B**) Flower plot illustrating target molecules pursued by major AI-driven pharmaceutical companies (e.g., Insilico Medicine, Compugen, Schrödinger, Lantern Pharma, HiFiBiO Therapeutics, and Relay Therapeutics). Target labels are color-coded by development phase: preclinical (light blue), clinical phase I (moderate blue), phase II (darker blue), and phase III (darkest blue). Shared and high-priority targets such as PD-1, PD-L1, TYK2, and EGFR are visible across companies and development stages.

Figure 4 comprehensively depicts the current focus and molecular strategies of AI-driven drug discovery initiatives. Figure 4A reveals a significant concentration of AI applications in oncology, which accounts for 126 studies (72.83%), far surpassing all other therapeutic areas. This outsized focus reflects the urgent unmet needs in cancer treatment and the availability of robust datasets in oncology, which are essential for training and validating AI models. The nature of cancer, characterized by genomic complexity and high inter-patient variability, makes it a fertile ground for AI applications that can sift through large-scale omics data, identify novel biomarkers, and propose tailored therapeutic strategies. Furthermore, oncology’s historically high rate of drug development investment likely incentivizes AI startups and pharmaceutical companies to focus on this domain, where the return on innovation can be particularly high. Beyond oncology, the distribution of studies across other disease areas shows a sharp drop. Dermatology ranks second with only 10 studies (5.78%), followed by gastroenterology and neurology with nine studies each (5.20%). These numbers indicate a nascent expansion of AI efforts into non-cancer areas, though the pace remains cautious. Dermatology, for example, benefits from the abundance of image-based data, which aligns well with AI tools such as convolutional neural networks. Neurology and gastroenterology, despite being rich in research potential, pose unique challenges such as limited biomarker availability and the complexity of brain–gut interactions, which might explain their relatively modest representation. Immunology accounts for seven studies (4.05%) and infectious diseases for five studies (2.89%), which gradually incorporate AI, particularly in areas like immune profiling, vaccine design, and antibiotic resistance prediction. Meanwhile, pulmonary and endocrinology have three studies each (1.73%), and rheumatology is the least represented with only 1 study (0.58%), underscoring their current underrepresentation, possibly due to insufficient data infrastructure or less immediate commercial incentive.

Figure 4B complements this therapeutic overview with a flower plot that maps the molecular targets pursued by leading AI-powered drug discovery companies. This visualization illustrates how AI is being used to expand therapeutic areas and diversify molecular strategies. The flower’s petals are divided among six prominent AI-driven biopharmaceutical firms: Insilico Medicine, Compugen, Schrödinger, Lantern Pharma, HiFiBiO Therapeutics, and Relay Therapeutics. Each sector’s molecular targets are placed according to their development phase, ranging from preclinical (light blue) to clinical phases I through III (in increasingly darker shades of blue). Notably, specific targets such as PD-1, PD-L1, TYK2, and EGFR appear in multiple petals, signaling their importance across company pipelines and suggesting convergence on high-value targets in immuno-oncology and precision medicine. The recurrence of targets like PD-1 and PD-L1, central to immune checkpoint inhibition therapies, indicates that AI is used to identify new molecules and optimize well-known mechanisms of action. Their widespread inclusion across companies and development stages signals intense competition and the strategic prioritization of validated but improvable pathways. Similarly, TYK2 and EGFR represent attractive targets with known therapeutic relevance in autoimmune and oncologic conditions [93,94], further reinforcing that AI enhances and accelerates drug discovery within proven biological frameworks. Additionally, more specialized or emerging targets, such as pan-TEAD, CDC7, and MALT1, demonstrate that AI is also facilitating novel hypothesis generation and target validation, which may yield first-in-class therapies.

## 3. Discussion

### 3.1. Main Findings and Comparison with Prior Works

This systematic review presents a comprehensive landscape of how AI is applied in the drug discovery and development (DDD) pipeline. The main findings reveal that AI adoption is predominantly concentrated in the early stages of drug discovery, specifically in target identification, validation, and compound screening, with a gradual extension into preclinical and early clinical development. Oncology emerges as the principal therapeutic area of AI activity, representing nearly 73% of identified studies, followed by dermatology, gastroenterology, and neurology. This distribution highlights a strategic focus by AI developers and pharmaceutical companies on high-burden, data-rich therapeutic domains. Additionally, AI technologies such as ML, DL, NLP, and RL were frequently reported, each employed according to task-specific requirements, such as image analysis, biomarker discovery, structure-based drug design, and patient stratification. This study aligns with and extends the existing body of knowledge compared to prior reviews in this domain. For instance, several findings highlighted the growing role of ML algorithms in streamlining target discovery and virtual screening [95]. However, these earlier reviews primarily focused on the promise of AI technologies, emphasizing algorithmic performance and proof-of-concept studies. In contrast, the present review integrates real-world implementation trends, industry partnerships, and clinical translation efforts, thereby offering a more grounded assessment of AI’s practical impact across the pharmaceutical value chain. Additionally, by disaggregating findings by disease area, development phase, and molecular strategy, this review reveals specific bottlenecks in underrepresented domains (e.g., rheumatology and endocrinology), which prior works only mentioned anecdotally.

One unique aspect of this review is its granular mapping of AI-driven pipelines at the company level, particularly through visualizations like the flower plot of molecular targets. This approach offers novel insights into how leading AI-powered biopharmaceutical firms prioritize molecular targets such as PD-1, PD-L1, EGFR, and TYK2, and how these targets are being pursued across different stages of development. While previous reviews acknowledged the theoretical ability of AI to identify novel targets, they rarely tracked how these insights translated into pipeline activities or advanced to clinical trials [96]. Identifying first-in-class targets (e.g., CDC7, pan-TEAD, and MALT1) further underscores AI’s growing role in hypothesis generation and mechanistic innovation, an aspect only sparsely covered in older literature. Moreover, this review distinguishes itself by explicitly analyzing global trends in AI-related research output, highlighting geographic disparities in AI adoption and institutional investment. The dominance of studies from the United States, China, and select European countries reflects a concentration of technical and financial resources, which may perpetuate regional inequalities in AI access and infrastructure.

### 3.2. Limitations and Future Works

Despite the systematic and rigorous methodology employed in this review, several limitations must be acknowledged that may impact the generalizability and scope of the findings. The review was constrained by strict inclusion criteria focusing exclusively on peer-reviewed studies reporting AI applications directly linked to drug development phases, specifically from target identification to preclinical and clinical development. While this approach ensured the quality and relevance of the included literature, it may have inadvertently excluded valuable insights from studies that addressed AI’s role more indirectly or those published in non-traditional formats such as preprints, white papers, or internal industry reports. Consequently, some potentially impactful AI applications or national initiatives, particularly from underrepresented regions, may not have been captured, leading to a potential selection bias.

The translational gap between AI model development and real-world clinical outcomes remains significant. While many studies demonstrated AI’s utility in early drug development stages, only a limited number progressed to report late-stage clinical outcomes or regulatory approvals. This highlights a critical need for future work to emphasize prospective validation, integration of real-world evidence, and regulatory harmonization to ensure the translational success of AI-derived drug candidates. Future research should focus on enhancing AI model transparency and interpretability to support regulatory decision-making and clinical trust. Additionally, integrating real-world data sources, such as electronic health records, biobank datasets, and patient-reported outcomes, could significantly enrich AI models and support more personalized, efficient therapeutic development. Moreover, collaborative international initiatives and shared infrastructure for data interoperability would help democratize AI capabilities across regions and reduce the concentration of innovation within select geographic and institutional hubs.

Another key challenge is the issue of data quality. Many AI models rely on high-throughput, multi-modal biomedical datasets that may be incomplete, biased, or inconsistently annotated. Noise or missing values can significantly affect model performance, generalizability, and replicability. Moreover, datasets used in training AI systems may not represent diverse patient populations, raising concerns about fairness and applicability across clinical settings. Ensuring standardized data curation protocols and robust data governance frameworks will be essential to improve the reliability of AI-driven drug discovery workflows. Another key limitation is the lack of standardized, quantifiable benchmarks to assess AI performance across drug development stages. There is no universally accepted set of key performance indicators (KPIs) for evaluating AI effectiveness in drug discovery. Metrics such as time-to-IND, lead optimization success rate, prediction accuracy for target-disease associations, and progression to clinical trials are rarely reported consistently across studies. Furthermore, outcome reporting is often narrative or descriptive, limiting objective cross-study assessment. This absence of uniform indicators complicates cross-platform comparisons and weakens the ability to assess domain-wide impact. Developing and adopting a consensus KPI framework tailored to drug discovery milestones would improve strategic evaluation and facilitate regulatory and clinical benchmarking.

From a methodological standpoint, future systematic reviews in this field should consider incorporating broader source databases, including gray literature and preclinical conference proceedings, to capture a wider range of AI applications and regional contributions. Standardizing data extraction frameworks and performance indicators, and incorporating quantitative meta-analytic techniques where feasible, could enhance comparability across studies. Moreover, employing living systematic review frameworks, where the literature is updated in real time, may be especially valuable in fast-evolving domains like AI in drug development, allowing continuous integration of emerging evidence and improving policy and research responsiveness.

## 4. Materials and Methods

### 4.1. Eligibility Criteria

This systematic review focused on identifying and analyzing scholarly and professional works that reflect the real-world application and downstream impact of AI in drug discovery and development. To ensure the relevance and rigor of the selected literature, only studies and reports demonstrating meaningful contributions to the AI-driven drug development pipeline were considered. Specifically, the review encompassed original research articles, comprehensive reviews, white papers, and technical or industry reports that detailed the use of AI in advancing one or more stages of the drug discovery process. These stages included, but were not limited to, target identification, compound screening, hit-to-lead optimization, and the progression of candidates into preclinical studies and clinical trials. Documents were prioritized if they described tangible outputs from AI-driven pipelines, such as successful candidate nominations, IND filings, or the initiation of clinical trials. Emphasis was placed on works that provided evidence of accelerated timelines, improved molecular profiling, or enhanced pipeline productivity resulting from AI integration. Conversely, studies were not considered if they focused solely on computational or algorithmic model development without any application to real-world pharmaceutical pipelines. Likewise, studies discussing AI applications in areas unrelated to the core drug discovery lifecycle, such as marketing, supply chain logistics, sales analytics, or pharmacovigilance, were excluded from consideration. This delineation ensured that the review remained centered on evaluating AI’s tangible contributions to therapeutic innovation, translational science, and clinical readiness.

### 4.2. Information Sources

Multiple information sources were systematically searched to ensure a comprehensive and representative collection of relevant literature. The primary bibliographic databases consulted included PubMed, Web of Science, Scopus, and Cochrane Library. These platforms were selected due to their extensive indexing of peer-reviewed journals and conference proceedings across the domains of biomedical sciences, pharmaceutical innovation, AI, and computational modeling. They offered broad and complementary coverage of academic research and industry-driven applications. In addition to scientific databases, the clinical trials registry ClinicalTrials.gov was queried to identify records of ongoing or completed trials involving AI-assisted drug development. This source provided insights into the translational impact of AI on candidate advancement and regulatory readiness, particularly those that have reached investigational or therapeutic stages.

### 4.3. Search Strategy

A comprehensive search strategy was developed to capture the literature at the intersection of AI and drug discovery and development. This strategy combined free-text keywords and controlled vocabularies, including MeSH terms in PubMed, to ensure broad and precise retrieval of relevant studies. The search queries were constructed around five core conceptual domains: (1) AI technologies (e.g., “artificial intelligence”, “machine learning”, “deep learning”, “neural networks”); (2) drug discovery and development stages (e.g., “target identification”, “compound screening”, “preclinical development”, “clinical trials”); (3) outcomes and impact metrics (e.g., “timeline reduction”, “pipeline productivity”, “time-to-IND”, “regulatory approval”); (4) industry and commercial adoption (e.g., “pharmaceutical industry”, “venture capital”, “strategic partnerships”, “AI integration”); and (5) AI-based pharmaceutical and biotechnology companies (e.g., “Insilico Medicine”, “BenevolentAI”, “Exscientia”, “Recursion Pharmaceuticals”, “Relay Therapeutics”, “Generate Biomedicines”, among others). Boolean operators (AND/OR) were used to link these concepts effectively, and advanced syntax such as truncation and phrase searching was applied to optimize term variation capture. A representative query included terms such as (“artificial intelligence” OR “machine learning”) AND (“drug discovery” OR “drug development”) AND (“timeline reduction” OR “pipeline performance”) AND (“pharmaceutical industry” OR “venture capital”) AND (“Insilico Medicine” OR “BenevolentAI” OR “Exscientia”).

Searches were executed across multiple bibliographic databases, including PubMed, Web of Science, Scopus, and the Cochrane Library. To supplement peer-reviewed sources, gray literature was identified through targeted searches in Google Scholar and manual retrieval of documents recommended by domain experts. Additionally, ClinicalTrials.gov was searched to identify ongoing or completed trials involving AI-developed drug candidates. Only English-language publications were considered. To enhance transparency and reproducibility, the complete search strategy, including specific database queries and applied search filters, is provided in Supplementary Data S2.

### 4.4. Study Selection

The study selection process was conducted using a two-phase approach designed to rigorously identify publications relevant to the application of AI in drug discovery and development. In the first phase, two independent reviewers screened the titles and abstracts of all retrieved records. This initial screening was guided by a predefined set of criteria, focusing on the relevance of AI methods to drug development pipelines and the presence of tangible outcomes. Records that clearly did not meet the criteria were excluded at this stage, while those deemed potentially relevant were moved to the next phase. During the second phase, full-text articles were reviewed in detail by the same two reviewers to assess their eligibility. This phase involved careful evaluation of the AI methodology employed, the specific application stage within the drug discovery pipeline, and the outcome measures reported. Studies that did not provide concrete results, such as candidate identification, IND filing, or progression into clinical trials, were excluded. Similarly, studies that applied AI to areas unrelated to drug discovery (e.g., diagnostic imaging, electronic health record analysis, or marketing) were also excluded. Discrepancies between reviewers were addressed through structured discussion; in cases where consensus could not be reached, a third reviewer provided arbitration to ensure objectivity. The selection workflow followed the Preferred Reporting Items for Systematic Reviews and Meta-Analyses (PRISMA) 2020 guidelines [97,98] to enhance methodological transparency and reproducibility. The PRISMA checklist can be seen in Supplementary Data S3. This review was not registered in PROSPERO or any similar systematic review registry. The primary reason for this is that PROSPERO currently prioritizes reviews with direct clinical outcomes and patient-related interventions, which may not fully align with the technology-driven and preclinical focus of our investigation. Nevertheless, we strictly followed PRISMA 2020 guidelines to maintain transparency and methodological rigor. Future related reviews with clinical endpoints will be considered for prospective registration to enhance traceability and reproducibility. Additionally, Table 2 summarizes the detailed criteria used to guide study inclusion and exclusion, which were aligned with the review’s objective of assessing AI’s impact on accelerating drug development timelines and improving productivity metrics across preclinical and clinical stages.

### 4.5. Data Extraction

A standardized data extraction form was meticulously developed and pilot-tested prior to formal data collection to ensure consistency and accuracy in capturing relevant information. The form encompassed a comprehensive set of variables, including bibliographic details (publication year, authors, journal, database), methodological features (study type, AI technique applied), and contextual information (drug discovery stage targeted, therapeutic area, geographic origin). Additionally, outcomes of interest were systematically recorded, such as key performance indicators (e.g., time-to-IND reduction, development timeline acceleration, clinical success rates), commercial outcomes (e.g., funding mechanisms, industry partnerships, licensing agreements), and any reported limitations. Two reviewers independently reviewed each article to minimize bias and discrepancies during data extraction. Any discrepancies were resolved through discussion and consensus between the reviewers. The data extraction framework also included binary indicators (Yes/No) for key metrics such as whether the study demonstrated timeline impact, reported clinical outcomes, or involved industry collaboration. This detailed approach allowed for systematically comparing AI applications across various stages of drug development and therapeutic areas.

### 4.6. Data Synthesis and Interpretations

The extracted data were synthesized using a qualitative narrative approach complemented by descriptive statistics. Studies were grouped based on the type of AI technique employed (e.g., DL, RL, ML), the stage of drug development targeted (e.g., target identification, lead optimization, preclinical or clinical phases), and the therapeutic area (e.g., oncology, neurology, infectious diseases). Frequency counts and proportions were used to summarize categorical variables such as AI method usage, therapeutic focus, and geographic origin. Key performance indicators, such as time-to-IND reduction, success rates, and commercial outcomes, were compared across studies to identify trends and outliers. Studies reporting similar metrics were narratively compared to highlight the magnitude of AI-driven impact on drug development efficiency and productivity. Where available, industry partnerships, external funding, and licensing agreements were also examined to assess real-world translational potential. The findings were interpreted in the context of methodological quality, generalizability, and limitations noted within the included studies. This synthesis enabled a multi-dimensional understanding of how AI technologies reshape modern drug discovery pipelines across research and commercial domains.

## 5. Conclusions

This systematic review demonstrates that AI is increasingly reshaping drug discovery and development by streamlining early-stage research, improving target identification, and enhancing decision-making across clinical phases. AI technologies, particularly ML, molecular modeling, and DL, have proven instrumental in accelerating compound selection, optimizing lead candidates, and supporting personalized therapeutic strategies. The current landscape shows a dominant focus on oncology, driven by data availability and commercial incentives, although applications are steadily expanding into other therapeutic domains. Despite the progress, a notable translational gap remains, as relatively few studies report clinical outcome data, indicating limited integration of AI-derived insights into later development stages. Industry partnerships and investment trends further highlight growing confidence in AI’s utility in pharmaceutical innovation. Future efforts should prioritize increasing AI model transparency, regulatory harmonization, and seamless integration with real-world data to enable broader clinical adoption. Improving data interoperability, enhancing model interpretability, and validating AI-generated outputs in clinical settings are equally critical. Additionally, expanding AI applications to underrepresented therapeutic areas and ensuring alignment with evolving regulatory frameworks will be essential for solidifying AI’s role as a core enabler of modern drug development.

## Figures and Tables

**Table 1:** Recent applications of AI techniques in drug discovery across therapeutic areas and development stages (2015–2025). This table presents data from a single study conducted by one pharmaceutical or AI company, showcasing the application of AI techniques in drug discovery and development across various therapeutic domains. The complete dataset, comprising 173 studies from multiple companies and research groups, is available in Supplementary Data S1.

Study; Year	AI Technique Used	Drug Discovery Stage	Therapeutic Area	Company/Funding Source
Dumbrava, EE. et al. [43]; 2024	Structure-based drug design, virtual screening, ADMET prediction, biomarker identification	Clinical Phase I	Oncology (TNBC, HGSOC, endometrial cancer with TP53 mutation/loss)	A2A Pharmaceuticals
Patel, MR. et al. [44]; 2024	ChemiRise, orbital virtual screening, intelligent SAR, Chemi-Net (AI-driven computational drug design, virtual screening, PK/PD prediction)	Clinical Phase I	Oncology (ER+/HER2- Breast Cancer)	Accutar Biotechnology Inc.
Niewiarowska, A. et al. [45]; 2017	BERG’s Interrogative Biology^®^ platform + Oak Ridge Frontier supercomputer: Bayesian AI modeling, Multi-Omics data integration, supercomputing	Clinical Phase II	Oncology (Pancreatic Cancer)	BERG LLC.
Xia, S. et al. [46]; 2022	DL for epitope mapping and functional screening, synthetic antigen design, multi-modal AI for antibody optimization	Preclinical	Oncology (HER2+ Breast Cancer)	Baseimmune
Molnar, J. et al. [47]; 2025	Knowledge graph and ML for target prioritization	Clinical Phase I	Gastroenterology (Ulcerative Colitis)	BenevolentAI
Hartman, G. et al. [48]; 2024	DNA-encoded library (DEL) screening, computational modeling, and structure–activity relationship (SAR) analysis	Preclinical	Immunology (NLRP3-related)	BioAge Labs
Risinger, R. et al. [49]; 2025	NovareAI: Drug repurposing via big data integration, ML-based target identification, predictive modeling for trial design	Clinical Phase Ib/II	Neuropsychiatry (Dementia/Agitation)	BioXcel Therapeutics
Rotta, M. et al. [50]; 2024	AI-driven FUSION™ System: Target ID, molecular modeling, PK/PD modeling, biomarker stratification	Clinical Phase I	Oncology (Acute myeloid leukemia/AML)	Biomea Fusion Inc.
Patel, J. et al. [51]; 2024	AI-based MAP platform for mutation analysis, allosteric site prediction, compound optimization, PK/PD modeling	Clinical Phase II	Oncology (Glioblastoma, non-small cell lung cancer/NSCLC)	Black Diamond Therapeutics
Idowu, O. et al. [52]; 2023	AI-driven epitope prediction, multi-omic integration, biomarker stratification, and predictive modeling via RAD platform	Clinical Phase I/II	Oncology (Advanced solid tumors)	Cancer Research UK
Grant, S. et al. [53]; 2023	ML (Target identification from scRNA-seq data via SCOPE platform)	Preclinical to Clinical Phase I	Gastroenterology (Ulcerative Colitis and Crohn’s Disease)	Celsius Therapeutics
Dumbrava, E. et al. [54]; 2021	Unigen™: ML–based predictive target discovery, AI-driven antibody design, spatial transcriptomics integration, and combination therapy modeling	Clinical Phase I	Oncology (Advanced Solid Tumors)	Compugen Ltd.
Khairnar, V. et al. [55]; 2023	Flex-NK™ platform: Computational antibody design, gene expression profiling, in vitro and in vivo modeling, combination therapy optimization using AI-driven analyses and structural modeling	Preclinical	Oncology (Multiple Myeloma)	Cytovia Therapeutics
Salto, MS. et al. [56]; 2024	Generative AI for compound design, RL for chemical space exploration, physics-based simulations for binding affinity optimization, automated synthesis and screening	Preclinical	Immunology (Rheumatoid Arthritis)	DeepCure Inc.
Xu, C. et al. [57]; 2023	IDInVivo platform: AI-driven in vivo gene targeting, preclinical efficacy modeling, PK/PD prediction, biomarker identification	Preclinical	Infectious Diseases (Hepatitis B)	Drug Farm
Wong, G [58]; 2024	AI-driven target discovery (Precision Insights), siRNA design (siRCH), pharmacokinetics modeling, biomarker-based stratification	Preclinical to Clinical Phase I	Pulmonary (Chronic Lung Disease)	Empirico
Khattak, A. et al. [59]; 2023	AI-Immunology™ platform (PIONEER™): Neoantigen prediction, ML, immune response modeling	Clinical Phase II	Oncology (Melanoma)	Evaxion Biotech
Diaz, N. et al. [60]; 2023	Generative design, ML for predictive modeling, simulation-guided clinical trial design	Clinical Phase I	Oncology (Renal cell carcinoma/RCC, NSCLC)	Exscientia and Evotec
Eckstein, F. et al. [61]; 2020	AI-assisted MRI segmentation and quantitative MRI (qMRI) analysis, location-independent cartilage change analysis, post hoc data analysis	Clinical Phase II	Rheumatology (Knee Osteoarthritis)	Formation Bio
Keating, AT. et al. [62]; 2024	AI-driven chemoproteomics, Druggability Atlas™ construction, covalent fragment-based drug discovery, ML, predictive modeling of resistance mechanisms	Clinical Phase I/II	Oncology (KRASG12C Mutant Tumors: NSCLC, PDAC, CRC)	Frontier Medicines
Wentzel, K. et al. [63]; 2024	GV20’s STEAD platform: AI-driven target discovery, antibody sequence prediction, and functional genomics integration	Clinical Phase I/II	Oncology (Advanced solid tumors)	GV20 Therapeutics
Guzman, B. et al. [64]; 2023	Magellan™ AI platform for allosteric modulator discovery, structural modeling, predictive modeling (PK/PD), biomarker identification	Preclinical	Neurology (Parkinson’s Disease)	Gain Therapeutics
Alwis, DD. et al. [65]; 2025	ML (Generate Platform) + iterative computation-experimentation loop	Clinical Phase I	Infectious Diseases (COVID-19 prophylaxis)	Generate Biomedicines
Spira, AI. et al. [66]; 2022	Generative AI, multi-modal predictive modeling, convolutional neural networks	Clinical Phase I	Oncology (Solid tumors including EBV+ gastric cancer, ccRCC, melanoma, mesothelioma)	HiFiBiO Therapeutics
Sanborn, RE. et al. [67]; 2024	Smart Allostery™ platform: AI-driven data mining, computational modeling	Clinical Phase I/II	Oncology (Advanced solid tumors)	HotSpot Therapeutics
Ahnert, JR. et al. [68]; 2023	RAD platform (AI-driven epitope prediction), mAbPredictAI (AI-guided antibody design), cross-species AI analysis, systems biology integration	Clinical Phase I	Oncology (TNBC, NSCLC, other solid tumors)	Hummingbird Bioscience
Adjei, AA. et al. [69]; 2024	Iambic AI: Physics-informed AI drug discovery platform	Clinical Phase I/Ib	Oncology (HER2-driven solid tumors)	Iambic Therapeutics
Ren, F. et al. [16]; 2025	Chemistry42: Generative models and RL	Clinical Phase III	Pulmonary (Idiopathic pulmonary fibrosis/IPF)	Insilico Medicine
Kim, H. et al. [70]; 2024	AI-driven secretome mining, quantitative proteomics, and phenotypic validation	Preclinical	Endocrinology (Diabetes Type 1)	Juvena Therapeutics
Leber, A. et al. [71]; 2023	LANCE^®^ AI Platform, TITAN-X AI Platform: ML, multiscale modeling, predictive analytics, and bioinformatics	Clinical Phase II	Gastroenterology (Ulcerative Colitis and Inflammatory Bowel Disease/IBD)	Landos Biopharma
McKean, W. et al. [72]; 2024	RADR^®^ AI platform for identifying DNA repair vulnerabilities, biomarker signatures, and mechanism of action of LP-284	Clinical Phase I/Ib	Oncology (Relapsed/Refractory B-cell NHL, Solid Tumors)	Lantern Pharma Inc
Huang, Y. et al. [73]; 2024	AiLNP (AI Lipid Nanoparticle) platform for lipid formulation optimization; AiTEM (AI Therapeutic Engine for mRNA) for mRNA therapeutic candidate optimization	Preclinical	Oncology (Hepatocellular Carcinoma)	METiS Pharmaceuticals
Wang, S. et al. [74]; 2024	DL, AI-based identification, and screening using IBM Watson	Preclinical	Infectious Diseases (Veterinary Bacterial Infections)	MIT and IBM Watson
Verstockt, B. et al. [75]; 2024	AI-powered precision medicine (TITAN-X Platform) for target discovery, biomarker identification, and trial optimization	Clinical Phase I/Ib	Gastroenterology (Ulcerative Colitis)	MedChemExpress, NIMML Institute
Khanna, D. et al. [76]; 2024	ML, QSAR models	Clinical Phase II	Dermatology (Skin diseases, autoimmune, fibrotic disorders)	Medi-Tate and Medidata AI
Hussain, A. et al. [77]; 2025	Schrödinger LiveDesign platform: Computational modeling, structural biology, ML	Clinical Phase IIa	Gastroenterology (Ulcerative Colitis)	Morphic Therapeutic, Schrödinger, Lilly
Leber, A. et al. [78]; 2025	TITAN-X Precision Medicine Platform: Gene expression analysis, Predictive modeling, multiomics data integration, mechanistic modeling, pharmacokinetic simulations	Clinical Phase I	Immunology (Systemic Lupus Erythematosus/SLE)	NImmune, MedPath, BioSpace
Wu, R. et al. [79]; 2024	neoBiologics™ and neoDegrader™ (AI for antibody design, protein degradation, PPI analysis, and immunogenicity prediction)	Preclinical to Clinical Phase I	Oncology (NSCLC, gastric, liver, esophageal tumors)	NeoX Biotech
Noel, MS. et al. [80]; 2024	Structure-based drug design, ML-based predictive modeling, medicinal chemistry optimization	Clinical Phase I/II	Oncology (Solid tumors)	Nimbus Therapeutics
Papadopoulos, KP. et al. [81]; 2025	AI-Driven Helicon design, computational physics integration, data science for trial optimization	Clinical Phase I/II	Oncology (Solid tumors)	Parabilis Medicines
Shin, DY. et al. [82]; 2024	AI-driven Chemiverse Platform: target identification, compound screening, ADMET Prediction	Clinical Phase I/II	Oncology (Acute Myeloid Leukemia)	Pharos iBio
Alfa, R. et al. [83]; 2024	Recursion OS: AI-driven drug discovery (DL, machine vision, predictive modeling, computational chemistry)	Clinical Phase I	Neurology (Cerebral Cavernous Malformations)	Recursion Pharmaceuticals
Schönherr, H. et al. [84]; 2024	Dynamo™ platform: Motion-based drug design (MBDD), molecular dynamics simulations, ML, AI-driven modeling	Clinical Phase I	Oncology (Solid Tumors, Intrahepatic Cholangiocarcinoma)	Relay Therapeutics
Gamez, J. et al. [85]; 2023	SOMAIPRO platform: AI-driven computational techniques to identify new mechanisms of action, predict drug-target interactions, and repurpose existing drugs for new indications	Clinical Phase II	Neurology (Huntington’s Disease)	SOM Biotech
Krueger, JG. et al. [86]; 2024	DL, molecular dynamics simulations, free energy perturbation (FEP)	Clinical Phase III	Dermatology (Psoriasis)	Schrödinger Inc.
Manasson, J. et al. [87]; 2024	IMPACT platform: ML-driven AI for IgG protease design, deimmunization (epitope elimination), pharmacokinetic/pharmacodynamic (PK/PD) modeling, and multi-mechanistic targeting for optimal drug performance	Preclinical	Immuno-oncology (Thrombocytopenia/ITP and Evans syndrome)	Seismic Therapeutic
Rao, S. et al. [88]; 2023	Pharma.AI: DL, RL, and generative chemistry	Preclinical	Oncology (Triple-negative breast cancer, B-cell non-Hodgkin lymphoma)	Shanghai Fosun Pharmaceutical Development Co. Ltd., Insilico Medicine
Dedic, N. et al. [89]; 2019	SmartCube^®^ platform (phenotypic screening, computer vision, ML)	Preclinical to Clinical Phase I	Neurology (Schizophrenia)	Sumitomo Pharma and PsychoGenics
Koblan, KS. et al. [90]; 2020	SmartCube^®^ platform (phenotypic screening, computer vision, ML)	Clinical Phase II	Neurology (Schizophrenia)	Sunovion Pharmaceuticals Inc.
Sowell, RT. et al. [91]; 2023	AI/ML-enabled target discovery, compound generation, and ADMET prediction	Preclinical	Oncology (Solid Tumors)	Supercede Therapeutics
Fakih, M. et al. [92]; 2024	DNA-encoded library screening, ML	Clinical Phase I	Oncology (Cancer)	Totus Medicines

**Table 2:** Inclusion and exclusion criteria were used in study selection. This table outlines the detailed eligibility criteria applied during the selection of studies included in this systematic review. The criteria were established a priori to ensure consistency and relevance in capturing studies focused on the application of AI in drug discovery and development.

Criteria Type	Inclusion Criteria	Exclusion Criteria
Study Type	Peer-reviewed original research articles, white papers, or technical reports focusing on AI-driven drug discovery or development	Editorials, opinion pieces, reviews, commentaries, preprints, general reviews without AI focus, or unverified gray literature
AI Method	Studies applying ML, DL, natural language processing, generative AI, RL, or knowledge graph-based models	Studies based solely on rule-based systems, deterministic algorithms, or expert systems without adaptive or learning capabilities
Application Focus	Application of AI in drug discovery pipeline stages: target identification, hit/lead optimization, compound screening, preclinical evaluation, IND submission	Studies focused on AI in diagnostics, radiology, electronic health records, hospital operations, marketing, or unrelated computational biology applications
Outcome Measures	Studies reporting on outcomes such as candidate nomination, time-to-lead, IND approval acceleration, development timeline reduction, or pipeline productivity	Studies lacking measurable outcomes or reporting only theoretical models without downstream drug development relevance
Language	Published in English	Published in languages other than English
Publication Date	Published between January 2015 and April 2025	Published before January 2015

## Data Availability

The original contributions presented in this study are included in the article/Supplementary Material. Further inquiries can be directed to the corresponding author(s).

## Supplementary Materials

The following supporting information can be downloaded at https://www.mdpi.com/article/10.3390/ph18070981/s1: Supplementary Data S1: Dataset of included studies; Supplementary Data S2: Full search strategy; Supplementary Data S3: PRISMA checklist.

## Abbreviations

The following abbreviations are used in this manuscript:

ADMET	Absorption, distribution, metabolism, excretion, and toxicity
AI	Artificial intelligence
AML	Acute myeloid leukemia
CV	Computer vision
DDD	Drug discovery and development
DL	Deep learning
GM	Generative model
HGSOC	High-grade serous ovarian cancer
HTS	High-throughput screening
IBD	Inflammatory bowel disease
IND	Investigational new drug
IPF	Idiopathic pulmonary fibrosis
KG	Knowledge graph
KPIs	Key performance indicators
ML	Machine learning
MMS	Molecular modeling and simulation
NLP	Natural language processing
NSCLC	Non-small cell lung cancer
OCD	Obsessive–compulsive disorder
OM	Omics integration
PBM	Physics-based modeling
PK/PD	Pharmacokinetics/pharmacodynamics
PRISMA	Preferred reporting items for systematic reviews and meta-analyses
QSAR	Quantitative structure–activity relationship
R&D	Research and development
RL	Reinforcement learning
SBDD	Structure-based drug design
SLE	Systemic lupus erythematosus
TNBC	Triple-negative breast cancer
