# Supplementary material for: From Lab to Clinic: How Artificial Intelligence (AI) Is Reshaping Drug Discovery Timelines and Industry Outcomes
Source: Pharmaceuticals (Basel). 2025 Jun 30;18(7):981. doi: 10.3390/ph18070981 (PMC12298131; doi:10.3390/ph18070981)
Supplement: Supplementary file 1 [file pharmaceuticals-18-00981-s001.zip › Supplementary Data S2 - Full search strategy.pdf]

Supplementary Data S2

Full Search Strategy

A. Search Terms and Query Construction

The search strategy was designed to thoroughly capture the intersection of artificial intelligence (AI) and drug discovery by incorporating both free-text keywords and controlled vocabulary (e.g., Medical Subject Headings [MeSH] in PubMed. The query focused on four main concepts: Artificial Intelligence Technologies, Drug Discovery and Development Stages, Outcomes and Impact Metrics, and Industry and Commercial Adoption.

1. Artificial Intelligence Technologies
   - Keywords: "artificial intelligence," "machine learning," "deep learning," "neural networks," "predictive modeling," "natural language processing," "AI-driven platforms," "artificial intelligence in drug discovery," "AI drug discovery models."
   - MeSH Terms: "Artificial Intelligence"[MeSH], "Machine Learning"[MeSH], "Neural Networks"[MeSH], "Data Mining"[MeSH], "Deep Learning"[MeSH], "Computational Biology"[MeSH].
2. Drug Discovery and Development Stages
   - Keywords: "drug discovery," "drug development," "target identification," "hit-to-lead optimization," "compound screening," "preclinical development," "IND application," "lead compound optimization," "clinical trials."
   - MeSH Terms: "Drug Discovery"[MeSH], "Drug Development"[MeSH], "Target Discovery"[MeSH], "Lead Compounds"[MeSH], "Preclinical Drug Evaluation"[MeSH], "Clinical Trials as Topic"[MeSH].
3. Outcomes and Impact Metrics
   - Keywords: "timeline reduction," "development cycle time," "pipeline productivity," "time-to-IND," "clinical success," "clinical approval," "regulatory approval," "acceleration of drug development."
   - MeSH Terms: "Time-to-IND"[MeSH], "Drug Approval"[MeSH], "Regulatory Approval"[MeSH], "Drug Development Acceleration"[MeSH], "Clinical Trial Outcome"[MeSH], "Predictive Modeling"[MeSH].
4. Industry and Commercial Adoption
   - Keywords: "pharmaceutical industry," "biotech," "venture capital," "strategic partnerships," "AI integration in pharmaceutical industry," "licensing agreements," "AI investment," "AI commercial adoption."

    - MeSH Terms: "Pharmaceutical Industry"[MeSH], "Biotechnology"[MeSH], "Licensing Agreements"[MeSH], "Industry Collaboration"[MeSH], "Venture Capital"[MeSH].
5. AI-Based Pharmaceutical and Biotechnology Companies
    - Keywords: "BenevolentAI," "Insilico Medicine," "Shanghai Fosun Pharmaceutical Development Co. LTD," "Recursion Pharmaceuticals," "Relay Therapeutics," "Schrödinger, Inc," "Morphic Therapeutic," "Generate Biomedicines," "Exscientia," "Evotec," "MIT and IBM Watson," "Medi-Tate and Medidata AI," "Evaxion Biotech," "Sumitomo Pharma & PsychoGenics," "Iambic Therapeutics," "Black Diamond Therapeutics," "Celsius Therapeutics," "HiFiBiO Therapeutics," "HotSpot Therapeutics," "Cancer Research UK," "Hummingbird Bioscience," "Landos Biopharma," "MedChemExpress," "NImmune," "Baseimmune," "BioAge Labs," "Biomea Fusion Inc.," "BioXcel Therapeutics," "Compugen Ltd," "Cytovia Therapeutics," "DeepCure," "Drug Farm," "Empirico," "Formation Bio," "Frontier Medicines," "Gain Therapeutics," "GV20 Therapeutics," "Juvena Therapeutics," "Lantern Pharma Inc," "METiS Pharmaceuticals," "NeoX Biotech," "Nimbus Therapeutics," "Parabilis Medicines," "Pharos iBio," "Recursion Pharmaceuticals," "Seismic Therapeutic," "SOM Biotech," "Supercede Therapeutics," "Totus Medicines."

The search query used in databases:

(("Artificial Intelligence"[MeSH Terms] OR "Machine Learning"[MeSH Terms] OR artificial intelligence OR machine learning OR deep learning OR neural network* OR AI-enabled OR AI-driven OR predictive modeling OR computational modeling OR reinforcement learning OR natural language processing) AND ("Drug Discovery"[MeSH Terms] OR "Drug Design"[MeSH Terms] OR drug discovery OR drug development OR lead optimization OR target identification OR compound screening OR hit identification OR pharmacological profiling OR preclinical development) AND (timeline* OR time factor* OR development time OR time reduction OR acceleration OR faster development OR productivity OR efficiency OR pipeline performance OR clinical outcomes OR trial optimization OR investigational new drug OR IND application OR regulatory approval OR success rate) AND ("Pharmaceutical Preparations"[MeSH Terms] OR pharmaceutical industry OR pharma trends OR commercialization OR licensing OR partnerships OR strategic alliances OR venture capital OR investment OR industry adoption) AND ("BenevolentAI" OR "Insilico Medicine" OR "Recursion Pharmaceuticals" OR "Relay Therapeutics" OR "Schrödinger" OR "Morphic Therapeutic" OR "Generate Biomedicines" OR "Exscientia" OR "Evotec" OR "GSK" OR "IBM Watson" OR "Evaxion Biotech" OR "Merck" OR "Sumitomo Pharma" OR "PsychoGenics" OR "Iambic Therapeutics" OR "Verge Genomics" OR "BERG" OR "Black Diamond Therapeutics" OR "Celsius Therapeutics" OR "HiFiBiO Therapeutics" OR "HotSpot Therapeutics" OR "Cancer Research UK" OR "Hummingbird Bioscience"

OR "Landos Biopharma" OR "MedChemExpress" OR "NImmune" OR "A2A Pharmaceuticals" OR "Accutar Biotechnology" OR "Baseimmune" OR "BioAge Labs" OR "Biomea Fusion" OR "BioXcel Therapeutics" OR "Compugen" OR "Cytovia Therapeutics" OR "DeepCure" OR "Drug Farm" OR "Empirico" OR "Formation Bio" OR "Frontier Medicines" OR "Gain Therapeutics" OR "GV20 Therapeutics" OR "Juvena Therapeutics" OR "Lantern Pharma" OR "METiS Pharmaceuticals" OR "NeoX Biotech" OR "Nimbus Therapeutics" OR "Parabilis Medicines" OR "Pharos iBio" OR "Seismic Therapeutic" OR "SOM Biotech" OR "Supercede Therapeutics" OR "Totus Medicines"))

B. Boolean Operators and Search Syntax

To combine the five key search concepts: Artificial Intelligence Technologies, Drug Discovery and Development Stages, Outcomes and Impact Metrics, Industry and Commercial Adoption, and AI-Based Pharmaceutical and Biotechnology Companies—Boolean operators (AND, OR) were strategically applied. Examples of how the search was constructed include:

- ("artificial intelligence" OR "machine learning" OR "deep learning") AND ("drug discovery" OR "drug development") AND ("timeline reduction" OR "time-to-IND") AND ("pharmaceutical industry" OR "venture capital") AND ("Insilico Medicine" OR "Recursion Pharmaceuticals" OR "Exscientia")
- Truncation was used to capture word variations (e.g., drug discover* retrieves "drug discovery," "drug discoveries") for broader inclusion of related terms.
- Phrase searching, by enclosing multi-word terms in quotation marks (e.g., "artificial intelligence", "drug development"), ensured precise retrieval of relevant literature.
- Company names were incorporated using a long OR clause to ensure the inclusion of studies mentioning notable AI-driven biotech and pharma firms.

Database	Query	Results
PubMed	(("Artificial Intelligence"[MeSH Terms] OR "Machine Learning"[MeSH Terms] OR artificial intelligence OR machine learning OR deep learning OR neural network* OR AI-enabled OR AI-driven OR predictive modeling OR computational modeling OR reinforcement learning OR natural language processing) AND ("Drug Discovery"[MeSH Terms] OR "Drug Design"[MeSH Terms] OR drug discovery OR drug development OR lead optimization OR target identification OR compound screening OR hit identification OR pharmacological profiling OR preclinical development) AND (timeline* OR time factor* OR development time OR time reduction OR acceleration OR faster development OR productivity	10,055

	OR efficiency OR pipeline performance OR clinical outcomes OR trial optimization OR investigational new drug OR IND application OR regulatory approval OR success rate) AND ("Pharmaceutical Preparations"[MeSH Terms] OR pharmaceutical industry OR pharma trends OR commercialization OR licensing OR partnerships OR strategic alliances OR venture capital OR investment OR industry adoption OR "Insilico Medicine" OR "BenevolentAI" OR "Recursion Pharmaceuticals" OR "Exscientia" OR "Relay Therapeutics" OR "Schrödinger, Inc" OR "Generate Biomedicines" OR "Evaxion Biotech" OR "Black Diamond Therapeutics" OR "HiFiBiO Therapeutics" OR "Hummingbird Bioscience" OR "Landos Biopharma" OR "NIMML Institute" OR "MedChemExpress" OR "NImmune" OR "Accutar Biotechnology" OR "Baseimmune" OR "BioAge Labs" OR "Biomea Fusion" OR "BioXcel Therapeutics" OR "Compugen Ltd" OR "Cytovia Therapeutics" OR "DeepCure" OR "Drug Farm" OR "Empirico" OR "Formation Bio" OR "Frontier Medicines" OR "Gain Therapeutics" OR "GV20 Therapeutics" OR "Juvena Therapeutics" OR "Lantern Pharma Inc" OR "METiS Pharmaceuticals" OR "NeoX Biotech" OR "Nimbus Therapeutics" OR "Parabilis Medicines" OR "Pharos iBio" OR "Seismic Therapeutic" OR "SOM Biotech" OR "Supercede Therapeutics" OR "Totus Medicines"))	
Web of Science	TS=("artificial intelligence" OR "machine learning" OR "deep learning" OR "neural network*" OR "AI-driven" OR "AI-enabled" OR "predictive modeling" OR "computational modeling" OR "natural language processing") AND TS=("drug discovery" OR "drug development" OR "target identification" OR "lead optimization" OR "compound screening" OR "hit identification" OR "preclinical development") AND TS=("timeline*" OR "development time" OR "time reduction" OR "acceleration" OR "efficiency" OR "pipeline productivity" OR "investigational new drug" OR "IND application" OR "regulatory approval" OR "clinical success") AND TS=("pharmaceutical industry" OR "biotech industry" OR "venture capital" OR "strategic	

	partnership*" OR "licensing agreement*" OR "investment trend*" OR "commercial adoption" OR "Insilico Medicine" OR "BenevolentAI" OR "Recursion Pharmaceuticals" OR "Exscientia" OR "Relay Therapeutics" OR "Schrödinger, Inc" OR "Generate Biomedicines" OR "Evaxion Biotech" OR "Black Diamond Therapeutics" OR "HiFiBiO Therapeutics" OR "Hummingbird Bioscience" OR "Landos Biopharma" OR "NIMML Institute" OR "MedChemExpress" OR "NImmune" OR "Accutar Biotechnology" OR "Baseimmune" OR "BioAge Labs" OR "Biomea Fusion" OR "BioXcel Therapeutics" OR "Compugen Ltd" OR "Cytovia Therapeutics" OR "DeepCure" OR "Drug Farm" OR "Empirico" OR "Formation Bio" OR "Frontier Medicines" OR "Gain Therapeutics" OR "GV20 Therapeutics" OR "Juvena Therapeutics" OR "Lantern Pharma Inc" OR "METiS Pharmaceuticals" OR "NeoX Biotech" OR "Nimbus Therapeutics" OR "Parabilis Medicines" OR "Pharos iBio" OR "Seismic Therapeutic" OR "SOM Biotech" OR "Supercede Therapeutics" OR "Totus Medicines")	
Cochrane Library	("artificial intelligence" OR "machine learning" OR "deep learning" OR "neural network*" OR "AI-enabled" OR "AI-driven" OR "predictive modeling" OR "natural language processing") AND ("drug discovery" OR "drug development" OR "target identification" OR "lead optimization" OR "hit identification" OR "compound screening" OR "preclinical development") AND ("timeline*" OR "development time" OR "pipeline productivity" OR "faster drug development" OR "IND application" OR "investigational new drug" OR "clinical trial design" OR "regulatory success") AND ("pharmaceutical industry" OR "commercial adoption" OR "venture capital" OR "industry partnership*" OR "investment trend*" OR "licensing" OR "Insilico Medicine" OR "BenevolentAI" OR "Recursion Pharmaceuticals" OR "Exscientia" OR "Relay Therapeutics" OR "Schrödinger, Inc" OR "Generate Biomedicines" OR "Evaxion Biotech" OR "Black Diamond Therapeutics" OR "HiFiBiO Therapeutics" OR "Hummingbird Bioscience" OR "Landos Biopharma" OR "NIMML	

	Institute" OR "MedChemExpress" OR "NImmune" OR "Accutar Biotechnology" OR "Baseimmune" OR "BioAge Labs" OR "Biomea Fusion" OR "BioXcel Therapeutics" OR "Compugen Ltd" OR "Cytovia Therapeutics" OR "DeepCure" OR "Drug Farm" OR "Empirico" OR "Formation Bio" OR "Frontier Medicines" OR "Gain Therapeutics" OR "GV20 Therapeutics" OR "Juvena Therapeutics" OR "Lantern Pharma Inc" OR "METiS Pharmaceuticals" OR "NeoX Biotech" OR "Nimbus Therapeutics" OR "Parabilis Medicines" OR "Pharos iBio" OR "Seismic Therapeutic" OR "SOM Biotech" OR "Supercede Therapeutics" OR "Totus Medicines")	
Scopus	(TITLE-ABS-KEY("artificial intelligence" OR "machine learning" OR "deep learning" OR "neural network*" OR "AI-enabled" OR "AI-driven" OR "predictive modeling" OR "computational modeling" OR "natural language processing")) AND (TITLE-ABS-KEY("drug discovery" OR "drug development" OR "target identification" OR "lead optimization" OR "compound screening" OR "hit identification" OR "preclinical development")) AND (TITLE-ABS-KEY("timeline*" OR "development time" OR "acceleration" OR "time reduction" OR "pipeline efficiency" OR "investigational new drug" OR "IND application" OR "regulatory approval" OR "clinical success")) AND (TITLE-ABS-KEY("pharmaceutical industry" OR "biotech industry" OR "venture capital" OR "investment" OR "strategic partnership*" OR "licensing" OR "commercial adoption" OR "Insilico Medicine" OR "BenevolentAI" OR "Recursion Pharmaceuticals" OR "Exscientia" OR "Relay Therapeutics" OR "Schrödinger, Inc" OR "Generate Biomedicines" OR "Evaxion Biotech" OR "Black Diamond Therapeutics" OR "HiFiBiO Therapeutics" OR "Hummingbird Bioscience" OR "Landos Biopharma" OR "NIMML Institute" OR "MedChemExpress" OR "NImmune" OR "Accutar Biotechnology" OR "Baseimmune" OR "BioAge Labs" OR "Biomea Fusion" OR "BioXcel Therapeutics" OR "Compugen Ltd" OR "Cytovia Therapeutics" OR "DeepCure" OR "Drug Farm" OR "Empirico" OR "Formation Bio" OR "Frontier	

	Medicines" OR "Gain Therapeutics" OR "GV20 Therapeutics" OR "Juvena Therapeutics" OR "Lantern Pharma Inc" OR "METiS Pharmaceuticals" OR "NeoX Biotech" OR "Nimbus Therapeutics" OR "Parabilis Medicines" OR "Pharos iBio" OR "Seismic Therapeutic" OR "SOM Biotech" OR "Supercede Therapeutics" OR "Totus Medicines"))	

C. Eligibility Criteria

Inclusion Criteria:

- **Original studies, reviews, white papers, or reports**: Studies that describe or evaluate the application of artificial intelligence (AI) in drug discovery or development. This includes research on AI-driven methodologies, AI-assisted drug discovery platforms, and case studies of AI applications in pharmaceutical R&D.
- **Outcomes from AI-based pipelines**: Studies that report on tangible outcomes from AI-based drug discovery pipelines, such as candidate nominations, IND filings, or clinical trial advancements.
- **Peer-reviewed studies**: Articles that are peer-reviewed and published in English-language journals from **1st January 2015 to 30th April 2025**. This period captures the rapid advancements in AI technologies and their integration into drug discovery over the last decade.

Exclusion Criteria:

- **Purely computational studies**: Studies that focus solely on computational modeling, simulations, or theoretical frameworks without demonstrating tangible outcomes like drug candidate nomination, preclinical testing, or clinical trials.
- **AI use in non-drug discovery areas**: Studies focused on AI applications in non-discovery areas, such as marketing, pharmacovigilance, regulatory compliance, or drug manufacturing, will be excluded as they do not align with the scope of the review.
- **News articles, opinion pieces, or blogs**: Publications that are not peer-reviewed, such as media articles, opinion pieces, and blogs, will be excluded from this review. These sources do not provide the robust scientific data required for the review.

D. Information Sources

The following databases and sources were utilized to retrieve relevant literature:

1. **PubMed**:

   A comprehensive resource for biomedical literature, particularly useful for identifying studies focused on AI applications in drug discovery and development within the clinical and biomedical domains.

2. **Web of Science**:

   A multidisciplinary database that includes articles, conference proceedings, and patents, ideal for a broader scope of AI-related research across various disciplines relevant to drug discovery.

3. **Cochrane Library**:

   A collection of systematic reviews and clinical trials focusing on healthcare and medical evidence is particularly beneficial for identifying reviews on AI's impact on clinical drug development processes.

4. **Scopus**:

   A large abstract and citation database for peer-reviewed literature, providing access to studies from various fields, including pharmaceutical research and AI technologies.

5. **ClinicalTrials.gov**:

   A database of clinical trials that includes information on trials incorporating AI technologies for drug discovery, optimization, or clinical testing phases.

E. Search Period and Updates

- **Search Period**:

  The search was conducted for studies published between **1st January 2015 and 30th April 2025** to reflect the rapid advancements in AI technology and its increasing integration into drug discovery pipelines. The year 2015 was chosen as a starting point due to significant developments in AI-driven drug discovery beginning around that time.

- **Search Updates**:

  The search will be updated periodically to ensure the inclusion of the most recent publications. Updates will be performed at **six-month intervals** during the review process to capture any new studies and breakthroughs that may emerge.

If significant new research is found, additional analyses may be incorporated into the review to ensure it remains current and comprehensive.
